# Transoesophageal ultrasound‐guided bronchoscopic aspiration of a superior mediastinal tumour using the BF‐UC290F instrument

**DOI:** 10.1002/rcr2.427

**Published:** 2019-04-10

**Authors:** Daisuke Minami, Yuki Takigawa, Hiroe Kayatani, Ken Sato, Keiichi Fujiwara, Takuo Shibayama

**Affiliations:** ^1^ Department of Respiratory Medicine National Hospital Organization Okayama Medical Center Okayama Japan

**Keywords:** BF‐UC290F, endoscopic, ultrasound‐guided, bronchoscopic fine‐needle aspiration, superior mediastinal tumour

## Abstract

A 46‐year‐old male with a superior mediastinal mass presented with a one‐month history of hoarseness and chest pain and was referred to our hospital. Although endobronchial, ultrasound‐guided, transbronchial needle aspiration (EBUS‐TBNA) was initially performed, we could not obtain an adequate specimen because of his severe cough and an inadequate EBUS view. During the same endoscopic session, we performed endoscopic, ultrasound‐guided, bronchoscopic fine‐needle aspiration (EUS‐B‐FNA) via a transoesophageal approach using the BF‐UC290F (Olympus, Tokyo, Japan), a third‐generation EBUS‐TBNA endoscope. The BF‐UC290F enabled smooth access through the oesophagus and a clear EBUS view of the mass, attributable, respectively, to the compact distal tip and the powerful angulation. Rapid on‐site cytology revealed that an adequate specimen had been obtained, and we terminated the procedure without inducing a severe cough. Histologically, the mass was a squamous cell carcinoma. EUS‐B‐FNA employing the BF‐UC290F was useful to diagnose the superior mediastinal mass.

## Introduction

Endobronchial, ultrasound‐guided, transbronchial needle aspiration (EBUS‐TBNA) appears to be at least as effective as mediastinoscopy for mediastinal staging of non‐small‐cell lung cancer but has a lower rate of complications, rendering the former procedure the initial investigation of choice in many institutions. If the tumour is located near or adjacent to the large airway, EBUS‐TBNA is diagnostically useful and safe 1. However, endoscopic, ultrasound‐guided, bronchoscopic fine‐needle aspiration (EUS‐B‐FNA) is recommended for patients with central lung tumours invisible on conventional bronchoscopy, provided that the tumours are located immediately adjacent to the oesophagus 2. The BF‐UC290F is a new, Olympus EBUS‐TBNA endoscope that is simple to control and that allows even difficult‐to‐reach lymph nodes and lesions to be accessed for staging and diagnosis 3. We present a patient with a superior mediastinal mass diagnosed by EUS‐B‐FNA using the BF‐UC290F after initial EBUS‐TBNA.

## Case Report

**Figure 1:**
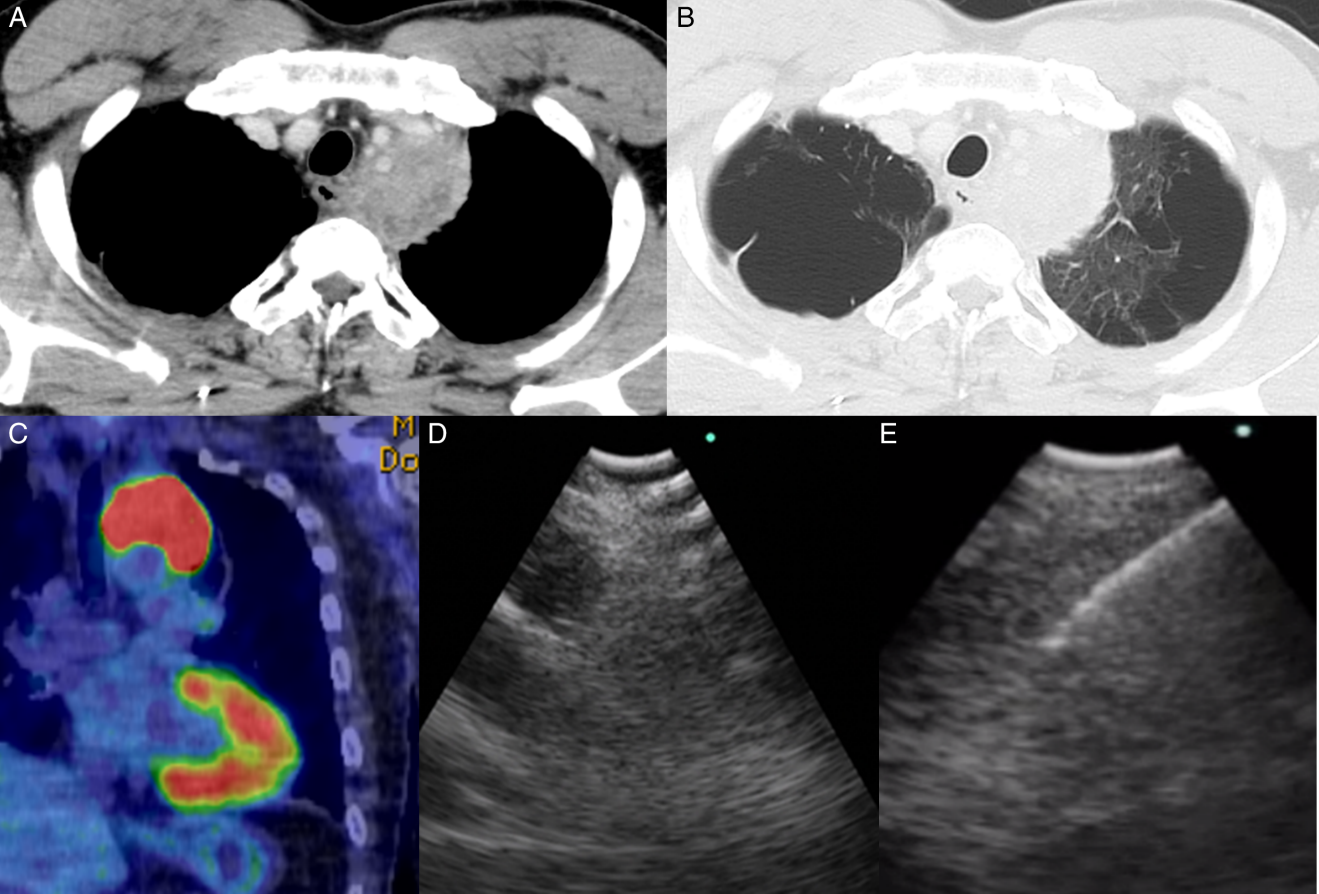
(A) Computed tomography revealed a superior mediastinal mass 60 mm in the longest diameter. (B) Emphysema was evident on computed tomography. (C) Fluorodeoxyglucose‐positron emission computed tomography revealed a hypermetabolic lesion at the location of the superior mediastinal mass. (D) The inadequate endobronchial image of the superior mediastinal mass. (E) The endo‐oesophageal image of the puncture procedure showing the needle within the superior mediastinal mass.

A 46‐year‐old male who had presented with a one‐month history of hoarseness and chest pain was referred to our hospital. He had no relevant medical history. On physical examination, chest auscultation revealed slightly diffuse expiratory rhonchi, but no other significant abnormality. His peripheral arterial blood oxygen saturation was 96% on room air. He was a current smoker. Chest computed tomography (CT) revealed a superior mediastinal mass (Fig. 1A) and pulmonary emphysema (Fig. 1B). Fluorodeoxyglucose‐positron emission CT revealed a hypermetabolic lesion at the site of the superior mediastinal mass (Fig. 1C); thus, we suspected a malignancy. The mass lacked the CT bronchus sign but lay adjacent to the trachea and the oesophagus.

**Figure 2:**
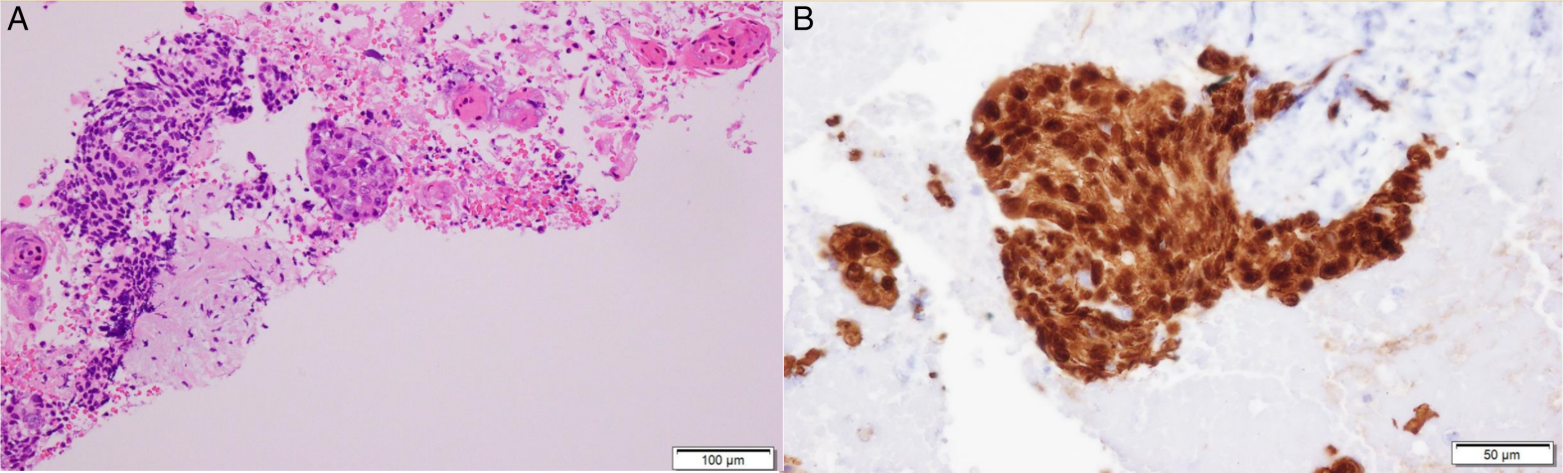
(A) The specimen obtained using a 22‐G ViziShot 2 needle during endoscopic, ultrasound‐guided, bronchoscopic fine‐needle aspiration was a squamous cell carcinoma. Haematoxylin and eosin (200×). (B) The cells were positive for p40 and cytokeratin 14 (CK14) (400×).

We initially chose EBUS‐TBNA for diagnosis. A total of 5 mL 2% (w/v) lidocaine was sprayed into the pharynx, and another 5 mL of 2% (w/v) lidocaine was administered via the channel during the procedure. A conventional flexible bronchoscope (BF‐F260; Olympus) was employed; intubation was achieved using a siliconized, uncuffed tracheal tube of internal diameter 7.5 mm (Portex; Smiths Medical, St. Paul, Minnesota, USA). A bronchoscope (BF‐UC290F; Olympus) was orally inserted during fentanyl‐ and midazolam‐induced conscious sedation. The patient received fentanyl (100 μg) and midazolam (8 mg) during the procedure. However, the EBUS‐TBNA approach failed because of his severe cough and an inadequate EBUS view (Fig. 1D). During the same endoscopic session, we performed EUS‐B‐FNA using a transoesophageal approach. The BF‐UC290F enabled smooth access through the oesophagus and a clear EBUS view of the mass, attributable (respectively) to the compact distal tip and the powerful angulation. He rarely coughed during the procedure. We identified a clear ultrasound image and punctured the mass with a 22‐G ViziShot 2 needle (Olympus) (Fig. 1E). Rapid on‐site cytology revealed that an adequate specimen had been obtained, and we terminated the procedure immediately. Histologically, the mass was a squamous cell carcinoma (Fig 2A, B). The stage was thus T4N0M0, and chemoradiotherapy was prescribed as first‐line treatment.

## Discussion

The BF‐UC290F, a third‐generation, reliable, Olympus EBUS‐TBNA endoscope 3 extends the applications of this line of instruments because it is compact (only 6.6 mm in outer diameter) and has a rigid region of only 25 mm, greatly improving manoeuvrability while maintaining a large 2.2‐mm‐diameter working channel that can accept the complete needle portfolio. EUS‐B‐FNA affords a high diagnostic yield, equivalent to that of EBUS‐TBNA, and has the advantages (compared to EBUS‐TBNA) of fewer oxygen desaturation episodes, less need for anaesthetics and sedatives, and a shorter procedural time 4. EUS‐B‐FNA should be considered in the same endoscopy session if bronchoscopy and EBUS‐TBNA fail 5. Initially, we could not obtain an adequate specimen because of the patient's severe cough and an inadequate EBUS view (Fig. 1D). One limitation associated with this case is that intubation tube disturbed the manipulation of the scope, particularly in angulation. EUS‐B‐FNA using the BF‐UC290F effectively diagnosed a superior mediastinal mass. The patient was a 46‐year‐old current smoker with a hypersensitive throat reflex despite administration of fentanyl 100 μg and midazolam 8 mg. He required no additional fentanyl or midazolam during EUS‐B‐FNA and we obtained an adequate specimen (without inducing severe coughing) via a transoesophageal approach. The BF‐UC290F features powerful angulation (to 160°), enhancing access to challenging sites, and improved puncture performance (a 5° steeper puncture angle than earlier instruments, allowing smooth penetration of the bronchial or oesophageal wall). The small size facilitated access through the oesophagus. To the best of our knowledge, this is the first report of a superior mediastinal mass diagnosed via EUS‐FNA using the BF‐UC290F. For this target, it might not be difficult to perform EUS‐B‐FNA even if they used the usual scope (BF‐UC260FW; Olympus). A larger‐scale study for a case which is difficult to access with conventional EBUS scope through the oesophagus would be necessary for emphasizing the usefulness of the BF‐UC290F during the EUS‐B‐FNA procedure.

In conclusion, EUS‐B‐FNA performed using the BF‐UC290F effectively diagnosed a superior mediastinal mass. EUS‐B‐FNA can be performed in the same endoscopy session if bronchoscopy and EBUS‐TBNA fail.

### Disclosure Statement

Appropriate written informed consent was obtained for publication of this case report and accompanying images.
